# The Mechanical Properties of Fiber Metal Laminates Based on 3D Printed Composites

**DOI:** 10.3390/ma13225264

**Published:** 2020-11-21

**Authors:** Bharat Yelamanchi, Eric MacDonald, Nancy G. Gonzalez-Canche, Jose G. Carrillo, Pedro Cortes

**Affiliations:** 1 Additive Manufacturing Research Center, College of Engineering, Youngstown State University, Youngstown, OH 44555, USA; pcortes@ysu.edu; 2 W. M. Keck Center for 3D Innovation, The University of Texas at El Paso, El Paso, TX 79902, USA; emac@utep.edu; 3 Unidad de Materiales, Centro de Investigacion Cientifica de Yucatan, 97205 Merida, Mexico; nancy.gonzalezcanche@gmail.com (N.G.G.-C.); jgcb@cicy.mx (J.G.C.)

**Keywords:** fiber metal laminate, 3D printing, impact, mechanical performance

## Abstract

The production and mechanical properties of fiber metal laminates (FMLs) based on 3D printed composites have been investigated in this study. FMLs are structures constituting an alternating arrangement of metal and composite materials that are used in the aerospace sector due to their unique mechanical performance. 3D printing technology in FMLs could allow the production of structures with customized configuration and performance. A series of continuous carbon fiber reinforced composites were printed on a Markforged system and placed between layers of aluminum alloy to manufacture a novel breed of FMLs in this study. These laminates were subjected to tensile, low velocity and high velocity impact tests. The results show that the tensile strength of the FMLs falls between the strength of their constituent materials, while the low and high velocity impact performance of the FMLs is superior to those observed for the plain aluminum and the composite material. This mechanism is related to the energy absorption process displayed by the plastic deformation, and interfacial delamination within the laminates. The present work expects to provide an initial research platform for considering 3D printing in the manufacturing process of hybrid laminates.

## 1. Introduction

Fiber metal laminates (FMLs) are structures based on alternating layers of metal and composite layers that are used as primary structures in the aerospace sector due to their unique mechanical performance under static and dynamic conditions [1]. Indeed, it has been shown that FMLs display outstanding impact and fatigue performance, as well as a remarkable environmental resistance [2]. However, one of the drawbacks of current FMLs is that their composite phase is based on thermosetting materials, which result in the need of relatively long curing cycles [3]. This inconvenience has been addressed by using thermoplastic composites, which can be adhered to the metal layers in a simple manufacturing step [4,5]. Additional advantages related to the use of thermoplastic based FMLs include the possibility of post-forming and repairing damaged parts in a short period of time [6,7]. In a study conducted by Reyes and Kang, it was shown that the self reinforced polypropylene based FMLs exhibited a tensile strength of 265 MPa, while the glass fiber reinforced polypropylene based FMLs exhibited a tensile strength of 254 MPa [8]. Guillen and Cantwell worked on determining the effect of cooling rate on the impact response and load carrying capacity of thermoplastic based FMLs and determined that while the composites and multidirectional FMLs are cooling rate dependent, the single directional FMLs are not [9]. Reyes and Gupta reported an interfacial fracture energy at around 1400 J/m^2^ and a tensile strength of 351 MPa and 464 MPa for hybrid FMLs with DP500 steel and polypropylene and glass fiber reinforced polypropylene [10]. This mechanical performance offered by the thermoplastic based hybrid laminates has intrigued the application of FMLs in the transportation sector, implying the incorporation of complex shapes, which requires the use of molds and elaborated manufacturing steps. Here, the production of customized geometries and performance could be addressed by processing these hybrid laminates through additive manufacturing.

The production of a printed composite that can provide unique mechanical properties under distinctive loading conditions can bring a degree of freedom to the design of structural parts. Across the different additive manufacturing (AM) technologies, fused filament fabrication (FFF) has become one of the most popular AM methods due to its low cost, versatility of materials, and ease of use [11]. FFF systems have been used to produce composites by incorporating reinforcing particles or short fibers to the plastic filaments to produce parts with better mechanical and thermal properties [12,13,14]. Although the incorporation of short fibers on printed materials enhances their tensile and flexural strength, it limits their dynamic response under low velocity impact conditions, due to the embrittlement of the parts [15,16]. On the other hand, the incorporation of a continuous fibers seems to enhance the overall mechanical performance of 3D printed structures. Matsuzaki et al. [17] investigated the production of continuously reinforced printed polylactic acid (PLA) parts using carbon and jute fibers with a fiber volume fraction of 6% and reported a superior tensile strength and modulus than when recorded on plain thermoplastic materials. Similar results have been reported on continuous, either carbon, glass or aramid fibers on PLA, nylon and acrylonitrile butadiene styrene (ABS) [18,19]. Additional studies have been conducted to further improve the FFF technology. Brenken et al. [20] have summarized the work performed on 3D printed continuously reinforced composites, and a tensile strength up to 256 MPa in carbon fiber reinforced PLA has been reported. Dickson et al. [21] also reported a tensile strength of about 206 MPa on 10% volume fraction of glass fiber reinforced nylon samples by adding expanding microspheres to the matrix.

In addition to the efforts on incorporating continuous reinforcing fibers on printed composites, the FFF technology has been considerably advanced by Markforged. They developed a robust continuous fiber 3D printer using carbon, glass, or aramid fibers [22]. Several studies of printed parts generated with Markforged have been characterized by different research groups and have reported attractive mechanical properties [23,24,25]. These results suggest that 3D printed composites can potentially be used as the reinforcing substrate on FMLs.

The present study has investigated the performance of an FML using a 3D printing continuous carbon fiber composite as the core component of the system. The manufactured laminates were evaluated under tensile, low velocity and high velocity impact conditions to characterize their quasi-static and dynamic performance. It is worth mentioning that the incorporation of carbon fiber with aluminum can result in galvanic corrosion condition due to the cathodic nature of the carbon fibers, a process previously addressed in the production of carbon reinforced aluminum laminates (CARE) [26,27]. However, the intention of the present work is to disseminate the novel implementation of 3D printing materials in FMLs to establish the technical platform for upcoming applications in the defense and transportation sector.

## 2. Materials and Methods

### 2.1. FML Elaboration

**Figure 1:**
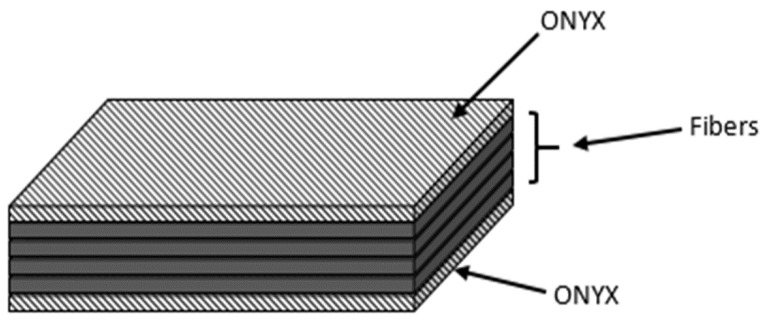
Schematic representation of the manufactured 3D printed composites.

In this work, a Mark Two^®^ 3D printer by Markforged (Watertown, MA, USA) was used to print continuous carbon fiber (CF) composite unidirectionally. The composite was based on six layers of printed materials with 0.125 mm layer thickness out of a 0.4 mm dual nozzle system. The four inner layers consisted of continuous carbon fiber printed at 250 °C and the external layers of a proprietary Markforged material known as ONYX printed at 272 °C, which is based on a mixture of chopped carbon fiber and nylon (see Figure 1). It is worth mentioning that the ONYX layers are integrated and printed in 45° orientation by default on any printing work performed by the Markforged printer.

**Figure 2:**
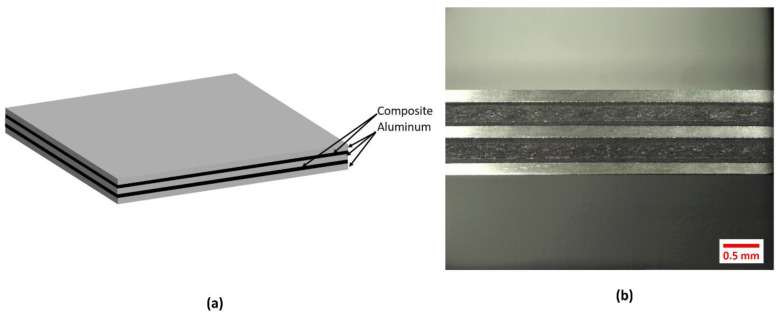
Investigated fiber metal laminates (FML) 3/2 system (**a**) Schematic representation (**b**) FML 3/2 side view.

The printed composites were subsequently incorporated between layers of aluminum alloy 2024-T3 to consolidate the FMLs (see Figure 2). The FMLs investigated were based on a 2/1 and 3/2 stacking configurations (see Table 1). Here, the first and second digit indicates the number of metal and composite layers, respectively.

The bonding between the metal and the composite was achieved by incorporating a 0.1 mm thick modified polyethylene film (Bemis 6343) at each metal-composite interface. All inner metal surfaces were slightly ground with a 180-grit sandpaper to improve the adhesion. After the grinding process, the metal was cleaned with isopropyl alcohol to remove all impurities. The materials stack was then placed in a square mold with inner dimensions of 10 × 10 cm^2^ with the metal rolling direction aligned in the same direction of the printed fibers.

The stacked system was then placed in a hot-press at 160 °C and 2 bars for 1 h. After this consolidation time, the heat was turned off under constant pressure until the sample reached room temperature. Once the FML sample reached room temperature, it was removed from the press and the mold. A micrograph of a FMLs here manufactured is shown in Figure 2. The composite volume fractions used for the FMLs were 43% and 50% for 2/1 and 3/2 arrays, respectively.

### 2.2. Tensile Test

The tensile tests were conducted on an Instron 5500R based on the ASTM D638/Type IV [28] on 100 mm length and 6 mm width FMLs, ASTM D3039 [29] for the composites with 100 mm length and 15 mm width, and ASTM E8 [30] for the plain aluminum alloy samples with 100 mm length and 6 mm width. In this work, at least three samples were tested on every system investigated. The load-displacement provided by the Instron unit was used to create nominal stress–strain graphs from every single tested sample. The nominal sample strain was calculated by dividing the cross head displacement by the gauge length.

### 2.3. Interfacial Fracture Toughness

The interfacial fracture toughness of the system was evaluated under quasi-static conditions using a 100 mm length and 20 mm width single cantilever beam (SCB) configuration [31]. An aluminum crack initiator was incorporated at 20 mm from end, in between the adhesive and the metal in the SCB specimens.

A steel fixture was used to clamp the SCB specimens at one end while the other end was loaded at a cross head displacement rate of 2 mm/min. The interfacial fracture energy, *Gc* was calculated using:

(1) $$G_{c}=\frac{P^{2}}{2b}\frac{dC}{da}$$

where *P* is the applied force, *b* is the specimen width, *C* is the specimen compliance, and *a* is the crack length. The specimen compliance in this case was determined from:

(2) $$C=c_{0}+ka^{3}$$

where *c_0_* and *k* are constants for a given specimen. Here, *k* was determined by measuring the slope of the graph of the compliance *C* versus the cube of crack length.

### 2.4. Low velocity Impact Test

**Figure 3:**
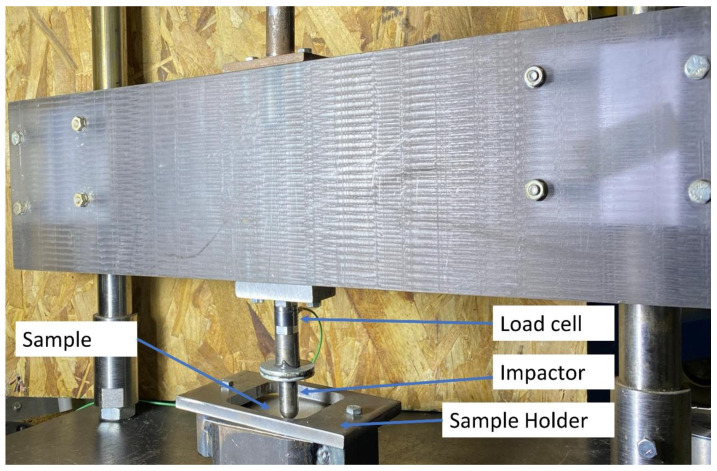
Impact tower and sample holder used to perform the low velocity impact tests.

Low velocity impact tests were conducted in a falling-weight impact tower, which was instrumented with a dynamic load cell Kistler of 50 kN, as represented in Figure 3. To induce different impact energies, impactor masses of 1.397 kg and 8.896 kg were dropped from different heights. The samples were clamped on an 80 mm diameter circular steel ring and impacted at their center by an impactor with a hemispherical geometry and a diameter of 15.7 mm. The tests consisted of performing impacts with energy increments and then correlating the changes observed in the force–time curves with the damage exhibited on each material. Table 2 shows the different conditions employed, including the impact energies used for each material.

### 2.5. High Velocity Impact Test

**Figure 4:**
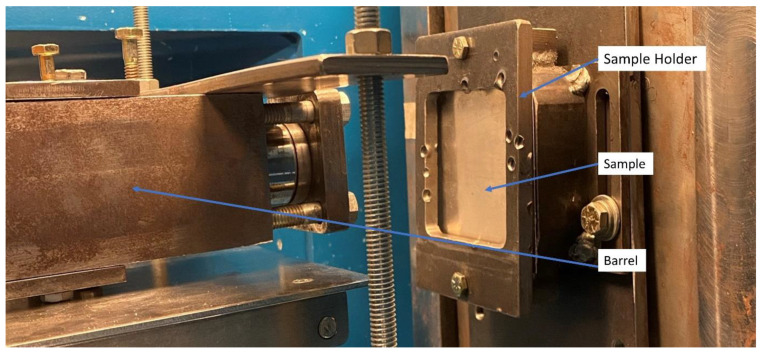
Gas gun apparatus used for performing the high velocity impact testing.

High velocity impact tests were conducted using a gas gun apparatus to study the dynamic deformation and perforation resistance of the studied materials (see Figure 4). The apparatus consists of three main components, i.e., pressure chamber, barrel, and sample holder. These kinds of tests are of significance to the aerospace sector since they simulate impact damage from hailstones and debris impacting on a plane fuselage [31]. Spherical stainless steel (SS) balls of 7.94 mm diameter with a mass of 2.05 g were used as projectiles on all the tested materials. The gas gun employed nitrogen as propellant connected to a two-meter barrel by a fast-acting pneumatic valve. The width–length dimensions of the tested parts were 100 mm × 100 mm. The thickness of each impacted system is displayed in Table 1. The samples were clamped in the gas gun, as shown in Figure 4. Here, a CED M2 shooting chronograph was used to measure the velocity of the SS projectile.

## 3. Results and Discussion

### 3.1. Tensile Test

**Figure 5:**
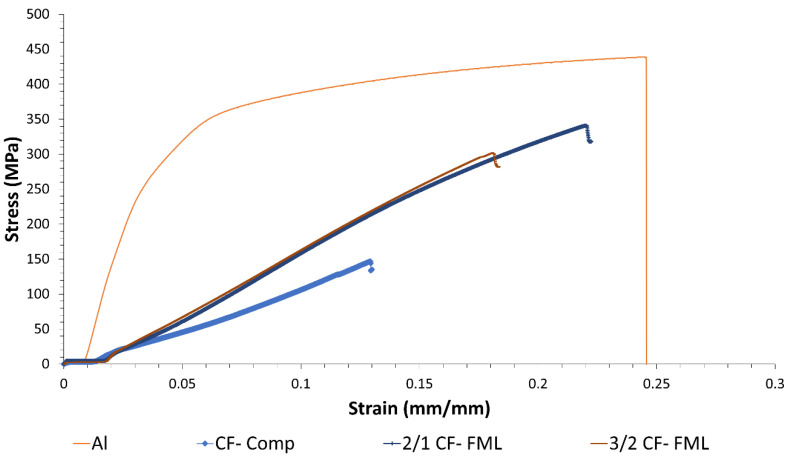
Representative tensile stress-strain curve of plain Al and carbon fiber (CF) FMLs.

Representative stress–strain curves of the tested materials are shown in Figure 5. From the figure, it is observed that the plain 3D printed composite and the FMLs appear to show a relatively linear response up to their ultimate tensile strength, followed by a sudden failure. In contrast, the plain aluminum alloy displayed a characteristic yielding point about 250 MPa followed by a ductile profile until it reached its ultimate tensile strength, which was about 440 MPa. From Figure 5, it is observed that the stress and strain of the FMLs seem to fall between the tensile properties of their individual components.

**Figure 6:**
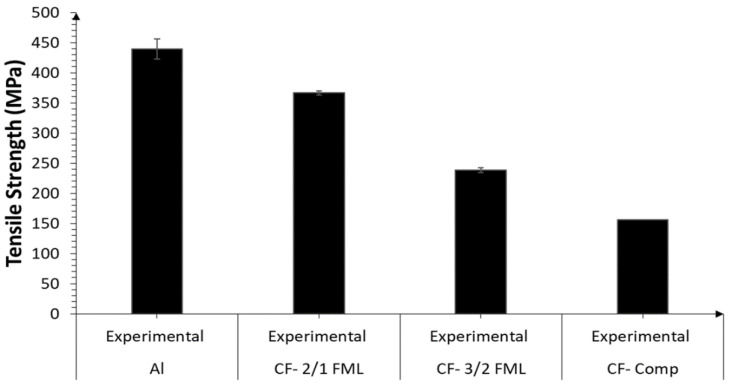
Tensile strength of the CF-FMLs and its constituent materials.

A summary of the tensile strength of the FMLs and the plain composite and alloy is shown in Figure 6. From the figure, it is observed that the plain aluminum alloy was about 20% and 87% higher than the FMLs 2/1 and 3/2, respectively. Indeed, the strength of the alloy resulted to be almost three times higher than that recorded on the plain composite. It is worth mentioning that the tensile strength of carbon fiber and ONYX materials used on the Markforged system have been reported to be about 800 and 36 MPa, respectively [32]. For the composite arrangement investigated here (four layers of carbon fiber and two of ONYX), a tensile strength of about 545 MPa would have been expected, using a simple rule of mixture. However, as previously mentioned, the investigated composite only had one single sheet of ONYX (0.125 mm thick) on each side of the four printed CF fibers. Thus, when the system was tested, these thin ONYX layers were not able to hold the splitting of the unidirectional CF fibers, resulting in a tensile strength considerably lower than that predicted using the data reported by Markforged [32].

**Figure 7:**
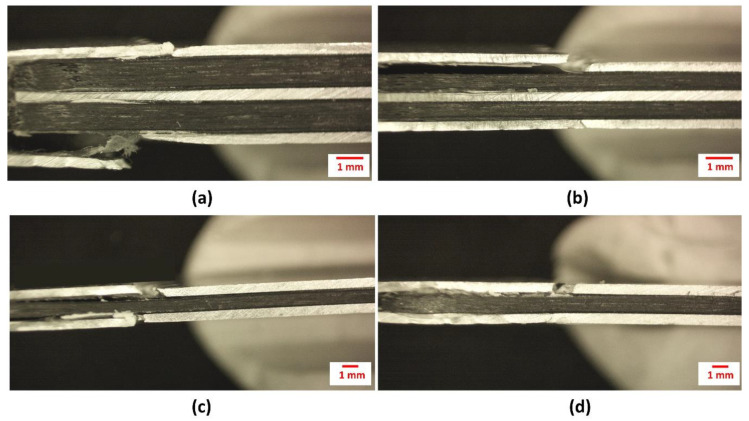
Tensile FML samples with (**a**,**b**) 3/2 configuration and (**c**,**d**) 2/1 configuration.

Besides the ability of the thin ONYX layer of holding the carbon fibers together during the tensile testing, some degree of delamination between the metal alloy and the composite was also observed, as shown in Figure 7.

**Figure 8:**
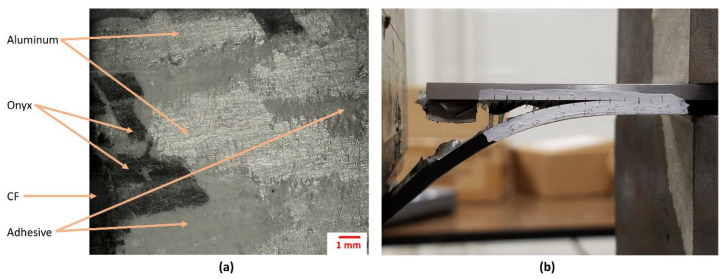
(**a**) Micrograph of the debonded face following the single cantilever beam (SCB) testing, where some residual ONYX and adhesive remained attached to the metal layer and (**b**) Sample during SCB testing.

The actual interfacial adhesion was evaluated here using the SCB testing geometry using Equation (1), and it was found that the average *G_c_* yielded about 1800 J/m^2^ (± 532.2) with an averaged k = 96.1 × 10^5^ (mm/N)/mm^3^. This interfacial fracture toughness seems to be lower than that reported on other thermoplastic based FMLs; where *G_c_* yielded values in the range of 2200 J/m^2^ to 2800 J/m^2^ [33,34,35,36]. This can be attributed to the lack of proper bonding between the ONYX and modified polyethylene adhesive as well as to an uneven adhesion between ONYX and the carbon fibers. Figure 8 shows the delaminated metal face following the SCB testing. From the figure; it can be observed that while some of the adhesive film remained attached to the metal—and a certain amount of the ONYX layer remained attached to the alloy—there were some large areas of the alloy that showed a bare-clean surface, indicating the need to further improve the metal–composite adhesion when using this 3D printed ONYX-CF composite. The lack of remaining fiber attached to the ONYX is also evident, indicating interlaminar fiber-ONYX delamination.

### 3.2. Low Velocity Impact Test

**Figure 9:**
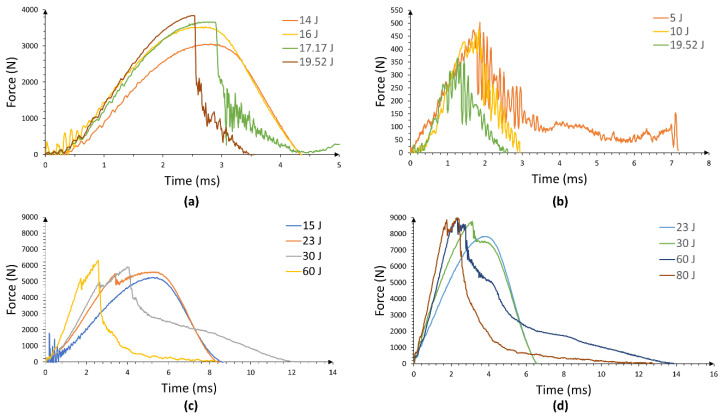
Force–time curves of FMLs and constituent materials for different impact energies (**a**) Plain aluminum, (**b**) Composite, (**c**) FML 2/1, and (**d**) FML 3/2.

**Figure 10:**
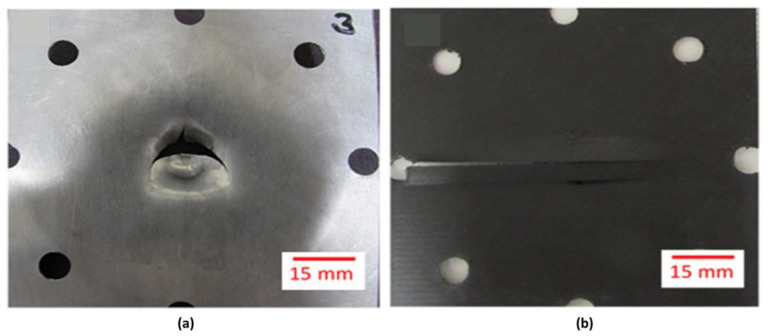
Perforation response of (**a**) Aluminum and (**b**) Composite specimens.

Figure 9a shows the force–time curves of the plain aluminum, evaluated at different impact energies. The force–time curves of 14 and 16 J correspond to the plastic deformation of the aluminum without producing the metal failure, while the curve of 17.17 J corresponds to the initial cracking of the aluminum. Finally, the curve of 19.52 J corresponds to perforation of the aluminum, as shown in Figure 10a.

Figure 9b shows the force–time curves of the composite for each impact energy evaluated, where the failure of the composite was observed as brittle. A mechanism that can be attributed to the nature of the fiber arrangement (unidirectional), which leads to fiber opening as the impactor hits the material. The force–time curves show an oscillating pattern that indicates the progress of brittle failure, exhibiting low peak forces of less than 500 N, which are much lower than the peak forces exhibited by the aluminum in all its curves. Figure 10b shows the perforated composite core, where the brittle failure is evident, with a crack running in the 0° fiber direction.

**Figure 11:**
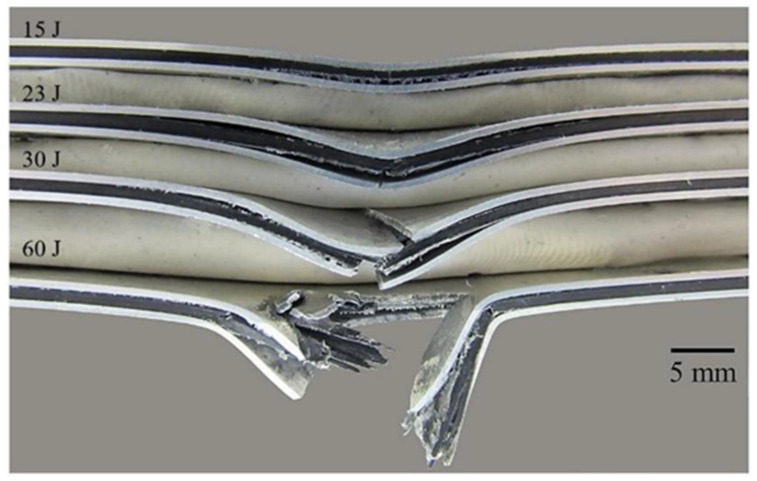
Cross sections of FML 2/1 at different impact energies.

Figure 9c,d shows the force–time curves of the FML 2/1 and 3/2, respectively (for each impact energy evaluated). It is worth noting that the peak forces for all curves are higher than the peak forces recorded on the aluminum and composite material. Here, the FMLs exhibited a greater load carrying capacity as the resulting combination of its constituents. In Figure 9c, the curve of 15 J corresponds to a plastic deformation response of the FML 2/1. Figure 11 shows the damage displayed by the FML 2/1, where composite–metal delamination, and composite fracture is observed. At 23 J, the sample shows a change in the impact behavior with a sudden force drop about 5500 N (see Figure 9c), which indicates the cracking of the aluminum skin at the non-impacted side, as shown in Figure 11, and delamination and cracking on the composites to a more extensive degree. The force–time curve of 30 J presents a more pronounced force drop behavior around 5000 N, which seems to correspond to the rupture of the aluminum layer in the non-impacted side (bottom side), as well as a second drop around 6000 N, which appears to be associated with the failure of the aluminum skin in the impacted side. Indeed, the remnant force indicates the breaking of the composite material. The force–time curve of 60 J shows the perforation behavior of the FML (see also Figure 11), where the fracture process included all the aforementioned failure mechanisms.

**Figure 12:**
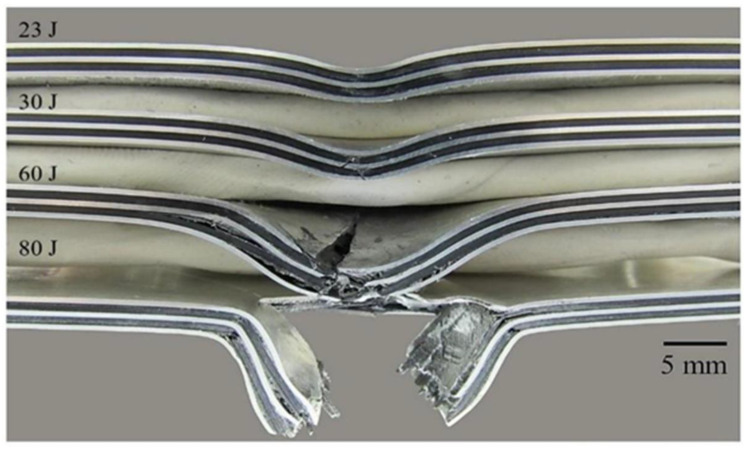
Cross sections of FML 3/2 at different impact energies.

The FML 3/2 was also subjected to low velocity impact conditions and its performance is shown in Figure 9d. The force–time curve of 23 J presents the plastic deformation response of the FML with an incipient failure in the localized impacted zone of the composite (see Figure 12). The force–time curve of 30 J presents a peak force with a force drop, related to the cracking of the aluminum skin in the non-impacted side and the intermediate aluminum layer, with the remnant of the displayed force related to the debonding between the composite and aluminum on the surrounding areas. The force–time curve of 60 J corresponds to the breaking of all layers in the FML with an extended metal-composite delamination. Lastly, the force–time curve of 80 J corresponds to the perforation of FML. Figure 12 shows the damage presented for the FML 3/2 at each impacted energy previously discussed.

**Figure 13:**
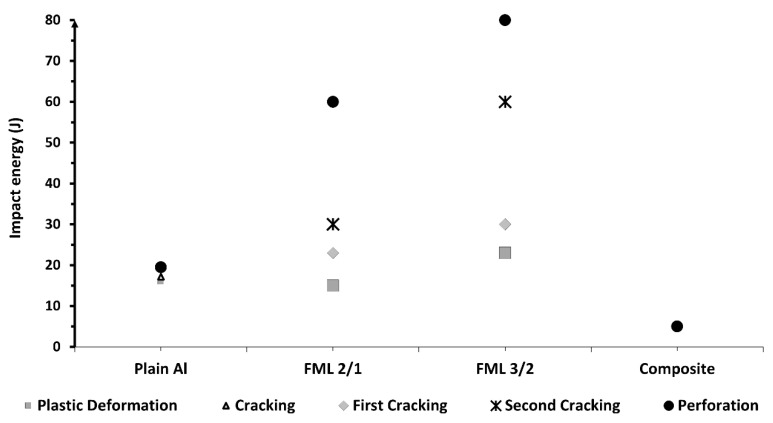
Impact energies for FMLs and constituent materials.

Figure 13 shows a comparison of the impact energy levels to produce the different damages observed for each material. It was found that for the plain aluminum, the energy levels for plastic deformation, cracking and perforation energy were close to each other, because plastic deformation and crack propagation were the only failure mechanisms involved here. In the thin composite material, a lower impact energy was recorded for perforating the system due to the brittleness of the composite and its low load carrying capacity. On the other hand, the FMLs show more spread impact mechanisms than the plain alloy and composite. This could be attributed to the different failure processes involved on these hybrid laminates. At 15 J the damage in the FML 2/1 presents plastic deformation, delamination, and the onset of cracking in the composite, while at 23 J, it produces the previous failure mechanisms and the fracture of the aluminum sheet at the bottom side. In contrast, at this impact energy (23 J) the FML 3/2 was only showing plastic deformation with an incipient damage in the composite. At 30 J, it was observed that while the FML 2/1 presents the second metal cracking on the impacted side, the FML 3/2 shows a full integrity in spite of an initial fracture at the intermediate and bottom metal layer, and the propagation of cracking in the composite material. At 60 J of impact energy, the FML 2/1 is perforated, while the FML 3/2 only exhibits the propagation of its aforementioned failure mechanisms. Lastly, the perforation of the FML 3/2 was achieved at 80 J, highlighting the higher energy absorption capacity of FMLs in comparison to their constituent materials when working as individual components.

**Figure 14:**
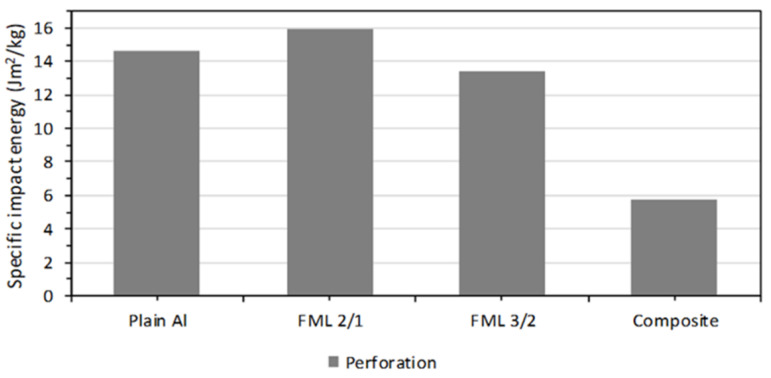
Specific impact perforation energies on the FMLs and the constituent materials.

In order to compare the impact performance of the FMLs and the constituent components, the specific impact perforation energies are presented here (see Figure 14). The composite presents the lowest specific perforation impact energy, which represents 39% of that displayed by the plain aluminum (14.67 Jm^2^/kg). From the figure, it is also observed that the FML 2/1 shows the highest specific perforation energy of all the systems investigated. The 2/1 configuration provided an impact performance 9% and 20% higher than the aluminum alloy and the FML 3/2, respectively. This behavior can be associated with the composite–aluminum delamination process that served as an energy dissipated mechanism. The figure also shows that the FML 3/2 system showed a specific impact energy 8% lower than the plain aluminum, a performance probably attributed to the increase in composite material, which seems to display a brittle-splitting behavior. Indeed, it appears that the large inclusion of composite volume fraction in the FML (about 50%) resulted in a decrease in the energy absorption capacity of these FMLs. These results suggest that the plastic deformation, the aluminum fracture, and the metal-composite delamination on FMLs seem to be the main fracture mechanisms for absorbing energy under low velocity impact conditions.

**Figure 15:**
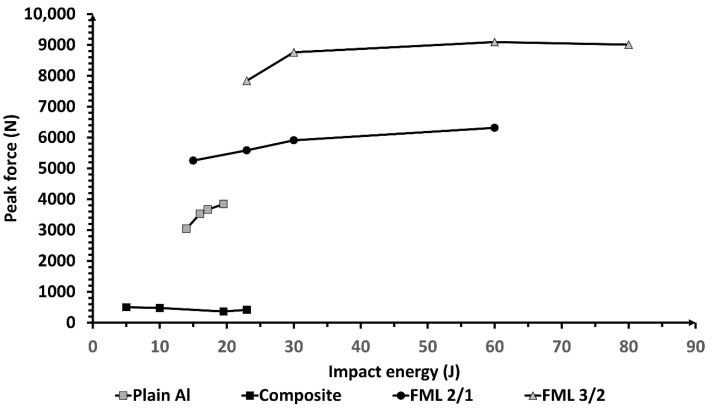
Peak force vs. impact energy of each studied material.

Figure 15 shows the peak force for each impact energy level evaluated in each material. Here, it is observed that FMLs present a higher load-carrying capacity than their constituent materials. It is observed that whilst the FML 3/2 exhibits the highest peak force, the composite displays the lowest. These results suggest that the FML 3/2 was strongly consolidated, although with a constrained displacement, due to the embrittlement and splitting failure mechanism of the composite.

### 3.3. High Velocity Impact Test

The high velocity impact tests were conducted using a gas gun and a summary of the results are shown in Table 3. From the table, it is observed that the highest perforation impact energy was displayed by the FML 3/2. It seems that under high velocity impact conditions, the strength provided by this arrangement creates a system capable of supporting higher impact velocities. In contrast, the plain composite shows the lowest impact perforation energy, a feature probably associated with the unidirectional arrangement of the composite. Indeed, unidirectional composites tend to show low impact properties due to a splitting mechanism during a high velocity impact event [37]. Included in Table 3, is the specific perforation energy of the materials investigated. From the table, it is observed that the plain aluminum alloy resulted in the highest specific impact performance. This result seems to be associated with the membrane effect, which has been reported for thin metals when impacted under high velocity conditions [37]. Here, the bending and stretching of the thin metal accounts for a high degree of energy absorption during the impact event.

**Figure 16:**
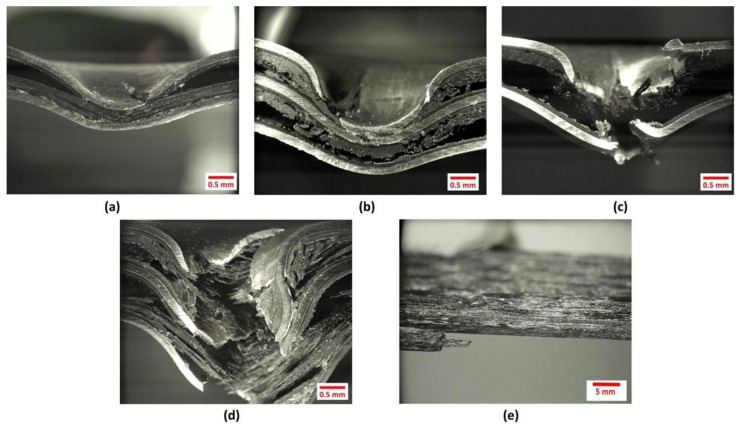
High velocity impacted samples. (**a**) FML 2/1 at 176 m/s (**b**) FML 3/2 at 273 m/s (**c**) Perforated FML 2/1 (**d**) Perforated FML 3/2 (**e**) Perforated plain composite.

Figure 16 shows the micrographs of the impacted FMLs under high velocity impact conditions. From the figure, it is observed that plastic deformation, interlaminar and interfacial delamination are the main fracture mechanisms displayed on the laminated structures. At an impact velocity below their perforation event (176 and 278 m/s on the FML 2/1 and 3/2, respectively), the samples show a plastic deformation on the top and bottom aluminum layers, as well as a visible fracture on the impacted layer. At these velocities, the bottom metal layers did not show visible cracks, and in the case of the FML 3/2, the intermediate metal layer did not present any cracks. Figure 16 also shows an intralaminar delamination on both FMLs, as well as a debonding between the fibers and the ONYX layer. It is observed that the ONYX layer remains attached to the aluminum sheet after the impact, suggesting that the adhesive material displays a strain-rate fracture mechanism. A process that has been observed on other metal–composite FMLs interfaces under high strain-loading rates [38].

The figure also shows micrographs of the perforated laminates. Here, it is observed that the FML 3/2 shows a larger plastic deformation and composite delamination than the FML 2/1. These features support the findings presented in Table 3, where the FML 3/2 displayed a superior impact performance than the FML 2/1. Included in Figure 16, is the micrograph of the impacted plain composite with a white arrow indicating the fiber printing orientation. A splitting feature was observed in the direction of the fibers. Indeed, this failure process barely provides enough energy absorption mechanism to dissipate the impact energy. A performance that supports the low perforation impact energy reported in Table 3.

## 4. Conclusions

The present work has investigated the mechanical performance of a new kind of fiber metal laminate (FML) constituted by a continuously 3D printed carbon fiber reinforced composite. Here, the tensile, low and high velocity impact performance of the laminates and their constituent materials have been evaluated. The initial results show that the tensile strength of the investigated FMLs are considerably superior to the plain composite materials. In contrast, the low velocity impact tests showed that the studied FMLs displayed a superior performance than that observed for their constituent materials. It was observed that the plastic deformation and the interlaminar delamination appear to be the principal energy absorption mechanisms on the laminates when subjected to impact events. Similar performance was observed in the high velocity impact tests, where the FMLs seem to surpass the impact performance of the plain composite materials. The present paper has investigated the use of 3D printing to produce hybrid laminates; an approach that can be further expanded into designed structures with unique and customized conformations and configurations. It is expected that this work can serve as an initial platform to keep further exploring the inclusion of additive manufacturing on high performance hybrid laminates. Current efforts are focusing on producing FMLs with strong and tough in-house 3D printed continuously reinforced composites, for matching those properties displayed by commercially available FMLs. The work will explore complex non-flat designs to highlight the benefits of additive manufacturing.

## Figures and Tables

**Table 1:** Stacking configurations investigated in this study.

Sample	Composite Volume FRACTION (%)	Thickness (mm)
Plain Al	0	0.5
FML 2/1 (Al/Comp/Al)	43	1.92
FML 3/2 (Al/Comp/Al/Comp/Al)	50	3.32
Plain Composite	100	0.77

**Table 2:** Impact energy evaluated for each investigated material.

Material	Areal Density (Kg/m^2^)	Impactor Mass (Kg)	Impact Energy (J)			
Plain Al	1.33	1.397	14	16	17.17	19.52
Plain Composite	0.87	1.397	5	10	19.52	-
FML 2/1	3.76	8.896	15	23	30	60
FML 3/2	5.95	8.896	23	30	60	80

**Table 3:** High velocity impact test results of the FMLs and its constituent materials.

Sample	Impact Velocity (m/s)	Perforation Impact Energy (J)	Specific Perforation Energy (Jm^2^/kg)
Plain Al	171	30.4	22.9
FML 2/1	204	42.6	11.3
FML 3/2	297	90.7	15.2
Plain Composite	21	0.45	0.5
